# One night of sleep deprivation induces release of small extracellular vesicles into circulation and promotes platelet activation by small EVs

**DOI:** 10.1111/jcmm.17528

**Published:** 2022-08-31

**Authors:** Chongyue Wang, Lulu Li, Chao Yang, Zhaoqiang Zhang, Xiao Li, Yun Wang, Xiang Lv, Xufeng Qi, Guohua Song

**Affiliations:** ^1^ School of Basic Medical Sciences The Second Affiliated Hospital of Shandong First Medical University & Shandong Academy of Medical Science Taian China; ^2^ Taishan Vocational College of Nursing Taian China; ^3^ Key Laboratory of Regenerative Medicine of Ministry of Education, Department of Developmental & Regenerative Biology Jinan University Guangzhou China

**Keywords:** extracellular vesicles, platelet activation, sleep deprivation

## Abstract

Extracellular vesicles (EVs) are emerging as key players in intercellular communication. Few studies have focused on EV levels in subjects with sleep disorders. Here, we aimed to explore the role of acute sleep deprivation on the quantity and functionality of circulating EVs, and their tissue distribution. EVs were isolated by ultracentrifugation from the plasma of volunteers and animals undergoing one night of sleep deprivation. Arterio‐venous shunt, FeCl_3_ thrombus test and thrombin‐induced platelet aggregation assay were conducted to evaluate the in vivo and in vitro bioactivity of small EVs. Western blotting was performed to measure the expression of EV proteins. The fate and distribution of circulating small EVs were determined by intravital imaging. We found that one night of sleep deprivation triggers release of small EVs into the circulation in both healthy individuals and animals. Injection of sleep deprivation‐liberated small EVs into animals increased thrombus formation and weight in thrombosis models. Also, sleep deprivation‐liberated small EVs promoted platelet aggregation induced by thrombin. Mechanistically, sleep deprivation increased the levels of HMGB1 protein in small EVs, which play important roles in platelet activation. Furthermore, we found sleep deprivation‐liberated small EVs are more readily localize in the liver. These data suggested that one night of sleep deprivation is a stress for small EV release, and small EVs released here may increase the risk of thrombosis. Further, small EVs may be implicated in long distance signalling during sleep deprivation‐mediated adaptation processes.

## 1 INTRODUCTION

Extracellular vesicles (EVs) are a heterogeneous group of cell‐derived membranous structures. They are present in biological fluids and are involved in multiple physiological and pathological processes. Extracellular vesicles are now considered as an additional mechanism for intercellular communication, allowing cells to exchange proteins, lipids and genetic material.^1^, ^2^ Peripheral blood contains a substantial amount of circulating EVs and their amount, composition and molecular profile reflects the physiological and pathophysiological condition of the body. Thus, EVs received recognition as potential biomarkers in semi‐invasive diagnostics.^3^ It is well documented that the number of EVs in the blood varies between individuals, and that EVs sub‐populations can originate from several tissues and are highly dynamic.^4^ In fact, the majority (about 25%) of blood‐derived EVs are thought to originate from the megakaryocytes, that is, either from circulating platelets or directly from platelet precursor cells, which reside in the bone marrow,^5^, ^6^, ^7^, ^8^ and, to a lesser extent, from erythrocytes, endothelial cells, lung epithelial cells and cardiomyocytes.^9^

Currently, EVs are commonly classified based on their intracellular origin. Thus, three principal populations of EVs are considered: apoptotic bodies, microvesicles (MVs) and exosomes.^10^ The apoptotic bodies (size between 50–5000 nm) are released by cells undergoing apoptosis and characterized by permeable membrane. MVs are directly shed from the plasma membrane of cells with a particle size of approximately 100–1000 nm.^10^ Exosomes (around 100 nm) are secreted from multivesicular endosomes.^10^ EVs act in cell‐to‐cell communication, delivering cargo from donor to recipient cells and modulating their physiological condition. In addition to a set of common EV proteins utilized for their characterization, EVs carry a pattern of biomolecules related to their mother cell. Their content includes protein, lipids, as well as nucleic acids, largely in the form of small RNAs. MV and exosome secretion occurs in constitutive and regulated fashion, controlled by Ca^2+^ signalling in response to extracellular signals such as ATP (monocytes), neurotransmitters (oligodendrocytes), depolarization (neurons), thrombin receptor activation (platelets), lipopolysaccharides (dendritic cells) or by cell stress.^6^, ^11^, ^12^, ^13^, ^14^, ^15^

Poor or insufficient sleep is a major public health problem affecting millions of people of all ages.^16^ Sleep deprivation and deficiency have a high prevalence in modern societies. The National Sleep Foundation reported that less than half (44%) of all Americans receive a good night's sleep almost every night.^17^, ^18^ According to the National Institute of Health, sleep deficiency is a broad concept that occurs (a) if an individual does not get enough sleep (sleep deprivation), (b) if an individual's sleeping habits are out of sync with the body's natural circadian rhythm (sleeping during the wrong time of the day), and (c) if the quality or quantity of sleep is diminished due to a sleep disorder or external factors.^18^ Therefore, there are mainly four specific variations of sleep deficiency: insomnia, acute total sleep deprivation (TSD), partial sleep deprivation (PSD) and night shift workers. PSD refers to the reduction in the total sleep time relative to one's usual baseline during a 24‐h period. PSD is the most common form of sleep deprivation encountered in everyday life in modern societies.^18^ Lack of sleep increases risk of obesity,^19^ diabetes,^20^ cancer^21^ and cardiovascular disease,^22^ but we know little about the underlying mechanisms that link sleep to disease. Given the fact that circulating EVs reflect physiological and pathophysiological conditions of the body and are of striking numbers on the order of 10^10^ ml^−1^ ^23^ and EVs have pro‐coagulant activity which may make the cases vulnerable to thrombotic or cardiovascular events,^24^ we aimed to explore the role of acute sleep deprivation on the quantity of circulating EVs, and then determine the functionality, including platelet activation and thrombotic events, of sleep deprivation‐induced EVs, and their subsequent tissue distribution.

## 2 METHODS

### 2.1 Subjects

All human studies were conducted in accordance with the *Declaration*‐*of*‐*Helsinki* and approved by the Human Ethics Committee of Shandong First Medical University. The identifier of Clinical Trial Registry was ChiCTR2000035490 (http://www.chictr.org.cn). All participants recruited from the graduate school students aged 23–28 years old provided written informed consent to participate before enrolment in the study. Exclusion criteria were diagnosed chronic cardiovascular or metabolic disease, psychiatric condition, sleep disorder or any other condition known to affect sleep including stressful condition; current or recent use of sleep medication (past 2 months). Descriptive characteristics of the participants are presented in Table 1. The subjects were asked to have habitual sleep 2 weeks before the experiment. In the sleep deprivation night, subjects were asked not to go to bed before 3 a.m. and not to wake up after 6 a.m. They were not allowed to consume caffeinated beverages and to smoke cigarettes before and during the task. Blood samples were collected before (7:00 a.m.) and after the sleep deprivation (7:00 a.m.).

**TABLE 1:** Subject characteristics (*n* = 20)

Characteristic	Mean ± SD
Age (years)	27 ± 1.24
Height (m)	1.63 ± 0.028
Weight (kg)	54.3 ± 3.29
BMI (kg/m^2^)	20.3 ± 0.57
Mean systolic BP (mmHg)	121.3 ± 3.39
Mean diastolic BP (mmHg)	83.6 ± 8.65

### 2.2 Experimental animals

Wistar rats and BALB/c‐nu nude mice were purchased from Beijing Weitong Lihua Experimental Animal Technology Co, Ltd. Wild‐type C57BL/6 mice were bred by the Institute of atherosclerosis, Shandong First Medical University. Age‐ and sex‐matched animals were used starting at 7–8 weeks of age. This study was approved by the ethics committee of Shandong First Medical University.

### 2.3 Establishment of one night of sleep deprivation animal models

For sleep deprivation (SD) studies, Wistar rats were placed in a sleep fragmentation chamber (Dichuangzhongshi). The sweep bar was shut off and stationary during the dark cycle (20:00–08:00), and the sweep bar moved along the bottom of the cage every 2 min during the light cycle (08:00–20:00). Sleep control (SC) rats that received undisturbed sleep were placed in sleep fragmentation chambers with stationary sweep bars. Blood samples were collected at 20:00.

### 2.4 Isolation and identification of plasma extracellular vesicles

Plasma extracellular vesicles were obtained by differential centrifugation as previously described with slight modifications.^25^, ^26^, ^27^ Briefly, cells and cell debris were gradually removed from plasma at the speed of 300 *g* 10 min and 2000 *g* 10 min. Then, platelets and apoptotic bodies were removed at 10,000 *g* 10 min. Based on others' experiences, the MVs can be clearly pelleted down at the minimum “*g*” forces of 20,000–30,000 *g* (2 times 30 min),^28^, ^29^ accordingly, the 10,000 *g* 10 min in our protocol can only settle down platelets and apoptotic bodies. Then, the supernatants were filtered through a 0.2‐μm membrane (Thermo Fisher Scientific) and transferred to the ultracentrifugation tube, centrifuged for 70 min at the speed of 100,000 *g* to retain the precipitate which contains small EVs and contaminating proteins. Then, the pellet was washed in PBS and centrifuged for another 70 min at the speed of 100,000 *g* to get small EVs. Each resultant pellet was finally re‐suspended in PBS buffer for use or stored at −80°C. The size and number of small EVs were determined by nanoparticle tracking analysis (NTA) with NTA analyzer (Zetaview). The small EVs were identified by transmission electronic microscope analysis. Briefly, small EVs were fixed with 2.5% glutaraldehyde at 4°C overnight. After washing, vesicles were loaded onto formvar‐coated grids, then negatively stained with aqueous phosphotungstic acid for 60 s and imaged with a transmission electron microscope (Jeol).

### 2.5 Western blot analysis

For EV marker protein analysis, the small EVs isolated from equal volumes of plasma of volunteers and rats were subjected to Western blot analysis which was performed according to the standard protocol as previously described.^30^ The following antibodies were used: rabbit anti‐HSC70 (Proteintech; 10645‐1‐AP; 1:3000), rabbit anti‐Alix (Sigma; ab186429; 1:5000), rabbit anti‐TSG101 (Proteintech; 28283‐1‐AP; 1:2000), rabbit anti‐CD63 (Sigma; ab216230; 1:1000), mouse anti‐Integrin αIIb (SC‐59923; Santa Cruz, 1:1000). For HMGB1 protein analysis, equal amounts of EV proteins (20 μg) were loaded and rabbit anti‐HMGB1 (Proteintech; 10829‐1‐AP; 1:2000) antibody was used.

### 2.6 Arterio‐venous shunt thrombosis model

The small EVs, isolated from equal volumes (2 ml) of plasma in individuals pre‐ or post‐SD and re‐suspended in 200 μl of PBS, were injected into WT rats by tail vein injection. Two hours after the injection, rats were anaesthetised with pentobarbital sodium (40 mg/kg body weight, i.p.) and fixed in a supine position. The toe pinch reflex, muscular relaxation and respiration rates were monitored to determine that adequate anaesthesia was administered. An arterio‐venous shunt tube was inserted between the left jugular vein and right carotid artery. The shunt was assembled from two 4‐cm polyethylene tubes (0.6‐ and 0.9‐mm inner and outer diameter, respectively) connected to a central tube (6‐cm long, 0.9‐mm inner diameter) containing a 5‐cm‐long cotton thread. The central tube was filled with NS and the 4‐cm polyethylene tubes were filled with a heparin saline solution (25 U/ml). Extracorporeal circulation was maintained for 15 min, and a thrombus adhered to the thread during this period. Then, the shunt was removed and the thread with its associated thrombus was withdrawn. The thrombus dry weight was determined 6 h later at room temperature by subtracting the weight of the dry 5 cm thread measured previously.^31^, ^32^

### 2.7 FeCl_3_ thrombus test

The small EVs, isolated from equal volumes (1 ml) of plasma in individuals pre‐or post‐SD and re‐suspended in 100 μl of PBS, were injected into WT C57BL/6 mice by tail vein injection. Two hours after the injection, a ferric chloride (FeCl_3_)‐induced carotid artery thrombosis model was established as described previously.^33^ Briefly, mice were anaesthetised with 1% Pentobarbital Sodium for 0.5–1 h to separate bilateral common carotid arteries. 5% FeCl_3_ solution was dripped on the filter paper, which covered the separated common carotid artery without damaging the surrounding tissues. After 15 min, the filter paper was removed, and the proximal common carotid artery was ligated. The common carotid artery was carefully cut‐off with scissors and immediately embedded with OCT (Japanese Cherry Blossom). Sections were stained with haematoxylin eosin. Observe and take pictures with IX51 microscope (Olympus).

### 2.8 Platelet aggregation test

To determine the effect of SD‐induced small EVs on platelet activation, we first prepared platelet rich plasma (PRP) from rats. In brief, after general anaesthesia, blood was collected from abdominal vein of male Wistar rats and anticoagulated with EDTA. The blood was centrifuged at 25°C for 10 min at 100 *g* to obtain the supernatant of PRP. Then, the blood was centrifuged at 25°C for 10 min at 1000 *g*, and platelet poor plasma (PPP) was obtained at room temperature. Then, the platelet count of each PRP sample was measured using an automated blood cell analyser (PE‐6800vet, Zibo) and adjusted to 2.5 × 10^8^/ml platelet by PPP. The effect of exosomes on thrombin‐induced platelet aggregation was performed using PRP prepared above and exosomes isolated from individuals pre‐ (pre‐exos) or post (pre‐exos)‐sleep deprivation. The maximum platelet aggregation rate was recorded within 5 min by using a LBY‐NJ4 platelet aggregation meter (Pulisheng). A total of 250 μl PRP was added to the reaction cup. Then, 1.75 μl of Thrombin (final concentration is 3.5 U), 15 μl of pre‐exos or post‐exos, isolated from equal volume of plasma (500 μl) of individuals, were added, respectively, and incubated at 37°C for 5 min. ADP (10 μl, 10 μM final concentration) or collagen (10 μl, 15 μg/ml final concentration) was used as agonist.

### 2.9 Intravital imaging of transplanted EVs

Donor Wistar male rats were randomly assigned to either SD or SC groups. One night of sleep deprivation rat models were established as described above. Both SC and SD rats were sedated by isoflurane inhalation and blood was collected from abdominal vein followed by euthanasia via cervical dislocation. Small EVs isolation from the plasma of SC or SD rats was carried out as described above and labelled in 1 mM lipophilic carbocyanine DiOC18(7) (DiR). 51 mg of washed, labelled small EVs from SC or SD donor rats were injected via tail vein into male, 8 weeks old BALB/c‐nu recipients. Recipient animals and untreated controls were sedated by 1% pentobarbital sodium and imaged at 8–12 h post injection by whole‐body intravital imaging on a Yena UVP chemstudio plus multifunctional imaging system (Yena). For tissue analysis, recipient mice were euthanized by cervical dislocation and tissues dissected and imaged by Yena UVP chemstudio plus multifunctional imaging system (Yena). Cryosections of 8 μm thickness were cut with a cryostat (Thermo Fisher) at −20°C and immediately perform fluorescence imaging by confocal microscopy (FV1200, Olympus). Fluorescence was detected using excitation and emission filters at 745 nm and 800 nm, respectively.

### 2.10 Statistical analysis

The normality of all values was conducted by using the Shapiro–Wilk test. The values of normality distributions were expressed as mean ± SEM. Median and interquartile range (IQR) were used for non‐normally distributed data. The differences between two groups for animal studies were tested by independent t‐test for normally distributed data and Mann–Whitney *U* test for non‐normally distributed. The differences between the pre‐ and post‐SD experiments were tested by paired *t*‐test for normally distributed data and Wilcoxon signed ranks test for non‐normally distributed. All of the statistical tests were performed with the GraphPad Prism software version 8.0, and *p* < 0.05 was considered to be statistically significant.

## 3 RESULTS

### 3.1 Acute SD triggers release of small EVs into the circulation

**FIGURE 1:**
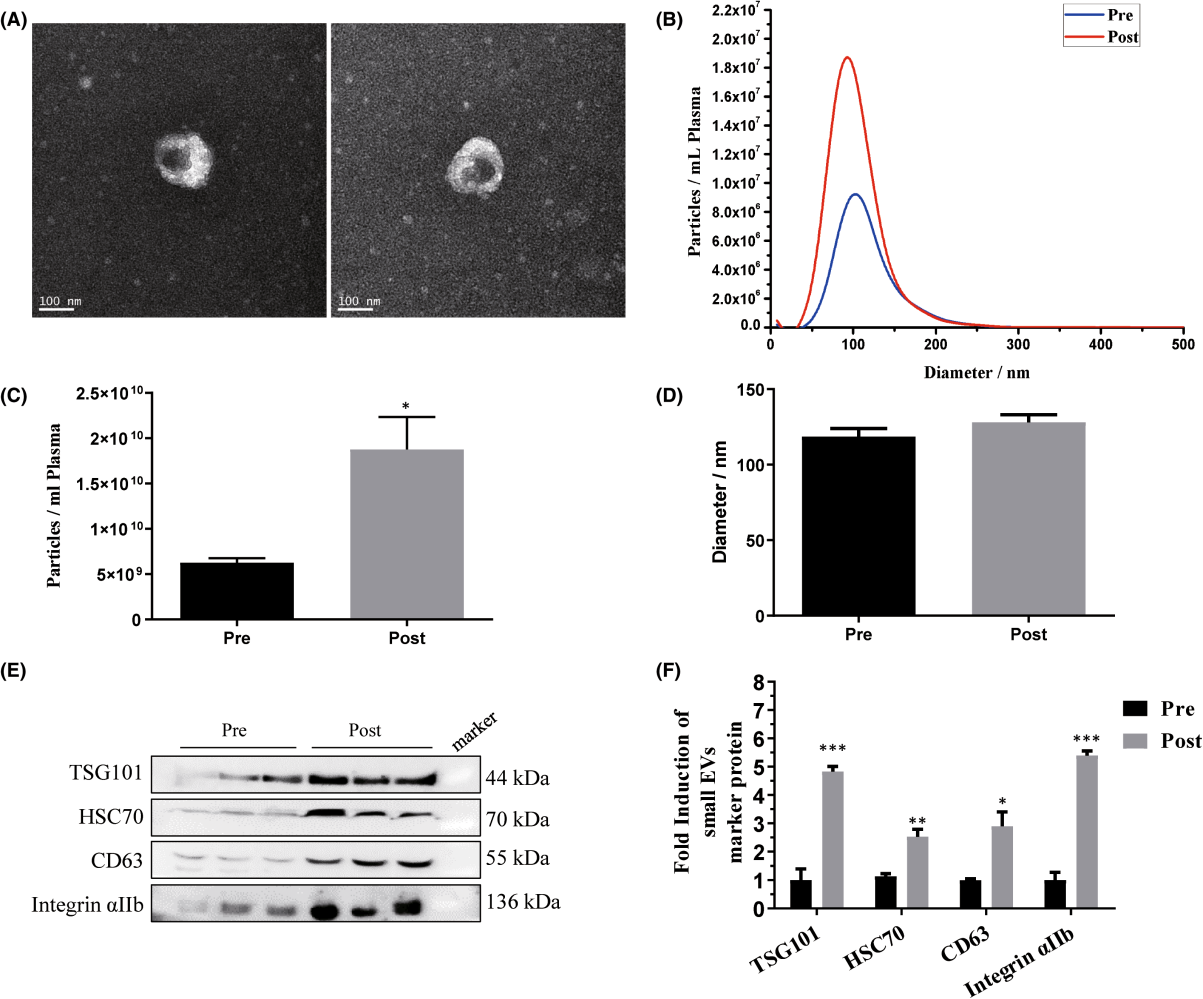
Effect of acute sleep deprivation on plasma small extracellular vesicles (EVs) in healthy participants. Plasma samples were collected from 20 healthy participants before (pre) and after (post) sleep deprivation, and 100,000 g pellets were prepared. (A) Particles were visualized by cryoelectron microscopy. (B–D) Particle concentration and size were measured by nanoparticle tracking analysis. *n* = 20. (E) Protein content of the particles was analysed by Western blotting (*n* = 6) and (F) the signals were quantified. Pre: pre‐sleep deprivation; Post: post‐sleep deprivation. Data are presented as mean ± SEM, *p < 0.05, **p < 0.01, ***p < 0.001.

To investigate the influence of one night of SD on the levels of circulating small EVs in plasma, we recruited 20 healthy (10 females, age 25 ± 5 years old) individuals who undergo one night of sleep deprivation. We collected venous blood samples (EDTA‐anticoagulated blood) before (pre) and after (post) SD, and prepared plasma. To analyse the levels of small EVs in the plasma samples, we performed differential centrifugation at 10,000 *g* removing platelet remnants and apoptotic bodies, followed by filtration of the supernatant and ultracentrifugation at 100,000 *g* to collect small vesicles of a size below 200 nm, which include exosomes (here defined as small EVs). Vesicles ~30–150 nm in diameter were observed by Transmission electron microscopy (TEM), which was consistent with previously reported characteristics of small EVs (Figure 1A). The collected small EVs were analysed by NTA revealing particles with a mean size of 120 (SEM ± 1.3) nm in diameter (Figure 1B,D). The total amount of particles increased in average 3.0 times directly after one night of SD (Figure 1C). Next, we investigated the small EVs of 6 subjects by Western blotting with antibodies against the EV marker proteins (Figure 1E) and quantified signal intensities (Figure 1F). The numbers of the 4 universal EV marker proteins increased on average 3.9 times after SD. It is worth mentioning that we detected integrin αIIb found on platelets indicating the presence and increment of platelet‐derived small EVs, based on the fact that the majority (about 25%) of blood‐derived EVs are thought to originate from the megakaryocytes.^5^

**FIGURE 2:**
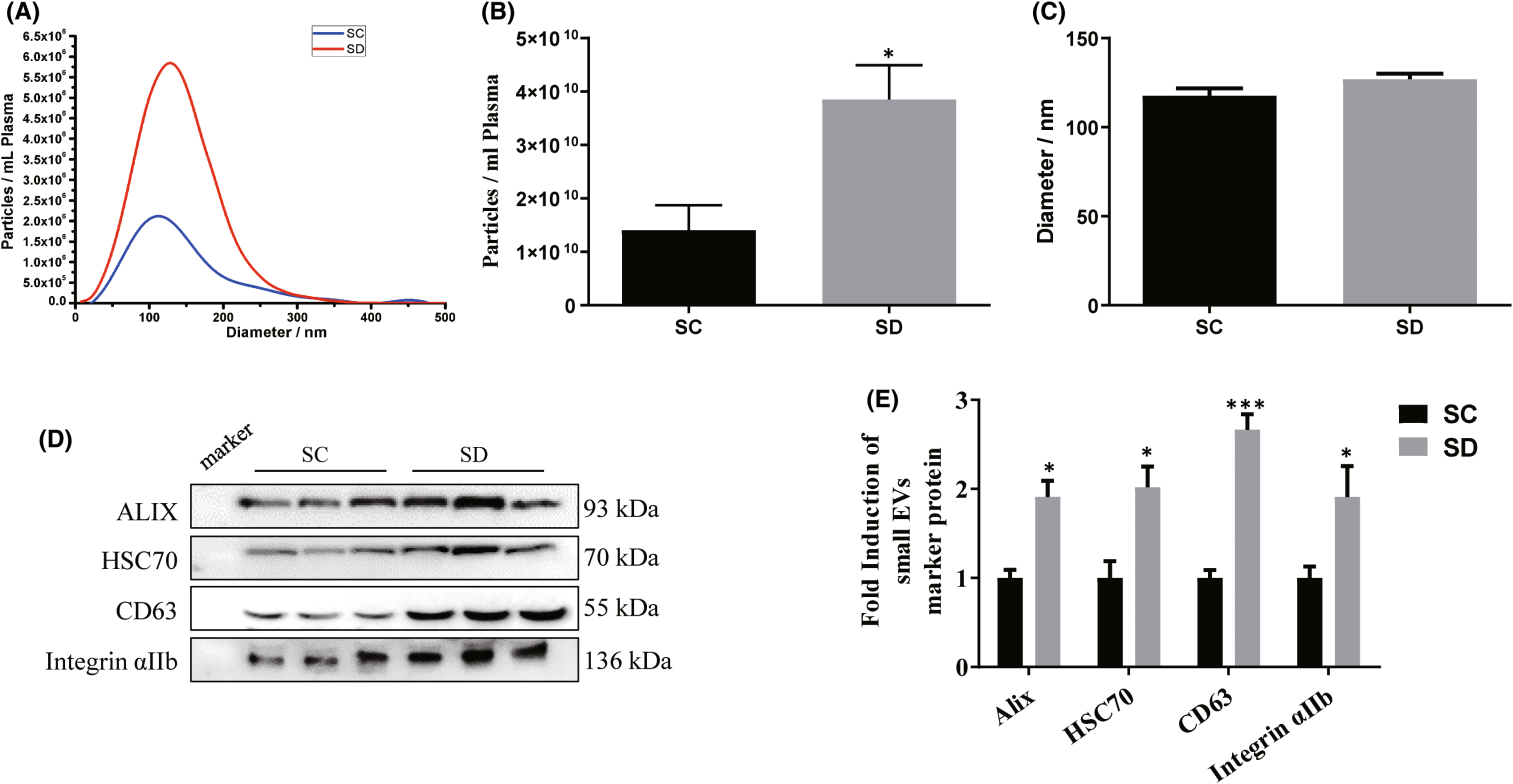
Effect of acute sleep deprivation on plasma small extracellular vesicles (EVs) in rats. Plasma samples were collected from rats carrying out one night of sleep deprivation and control group, and 100,000 g pellets were prepared. (A–C) Particle concentration and size were measured by nanoparticle tracking analysis. *n* = 10. (D) Protein content of the particles was analysed by Western blotting (*n* = 6) and (E) the signals were quantified. SC, Sleep control group; SD, Sleep deprivation group. Data are presented as mean ± SEM, *p < 0.05, ***p < 0.001.

Next, we performed controlled animal experiments in rats to further confirm the effect of SD on the amount of small EVs in the circulation. As shown in Figure 2A,B, the amounts of small EVs increased in average 2.7 times in SD group compared with SC control group, and particle size was not altered in 2 groups (Figure 2C). The EV marker proteins increased on average 2.1 times in SD group compared with control (Figure 2D,E). The data was in consistent with human study and indicates that acute SD leads to an increase in circulating small EVs.

### 3.2 Injection of sleep deprivation‐liberated small EVs increased thrombus formation and weight in thrombosis models

**FIGURE 3:**
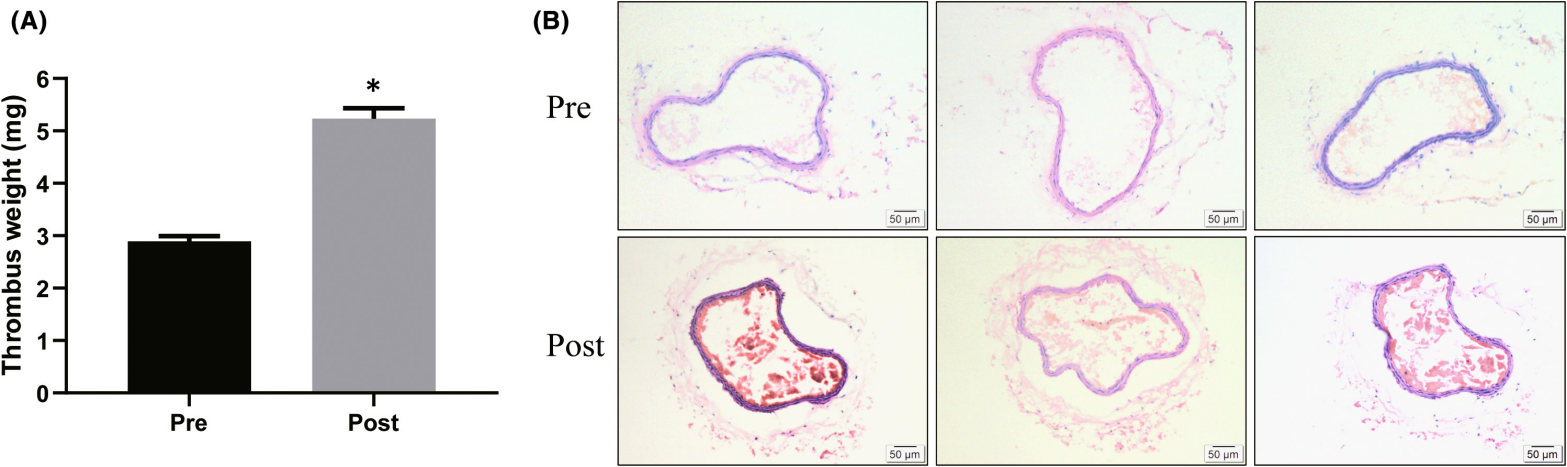
Effects of sleep deprivation‐liberated small EVs on thrombus formation and weight in thrombosis models. Blood samples were collected before (pre) and after (post) SD from 8 participants, and isolated small extracellular vesicles (EVs). These small EVs were then intravenously administered to recipient rats and mice. (A) Thrombus weight in a rat arterio‐venous shunt thrombosis model, *n* = 8. (B) Thrombosis formation in a ferric chloride‐induced arterial thrombosis model in mice, *n* = 8. Pre: pre‐sleep deprivation; Post: post‐sleep deprivation. Data are represented as means ± SEM, *p < 0.05.

Sleep disruptions were associated with prothrombotic changes and might be an independent risk factor for arterial thrombosis, which is associated with high cardiovascular morbidity and mortality.^34^ Here, we investigated the effects of SD‐liberated small EVs on thrombosis. We collected venous blood samples before (pre) and after (post) SD from 8 participants, and isolated small EVs. Pre‐exos and post‐exos isolated from equal volumes of plasma were then intravenously administered to recipient animals. First, an arteriovenous shunt thrombosis model was used. As shown in Figure 3A, tail vein injection of SD post‐liberated small EVs resulted in an increased thrombus dry weight compared with the control group injected with pre‐liberated small EVs. Next, in order to further examine in vivo prothrombotic activities of SD‐liberated small EVs, the FeCl_3_‐induced arterial thrombosis model was used, which is sensitive to antiplatelet drugs.^35^ As shown in Figure 3B, the formation of thrombus is increased in SD‐liberated small EV group. These data suggested that sleep deprivation‐liberated small EVs increased thrombus formation and weight in thrombosis models.

### 3.3 Sleep deprivation‐liberated small EVs promoted platelet aggregation induced by thrombin

**FIGURE 4:**
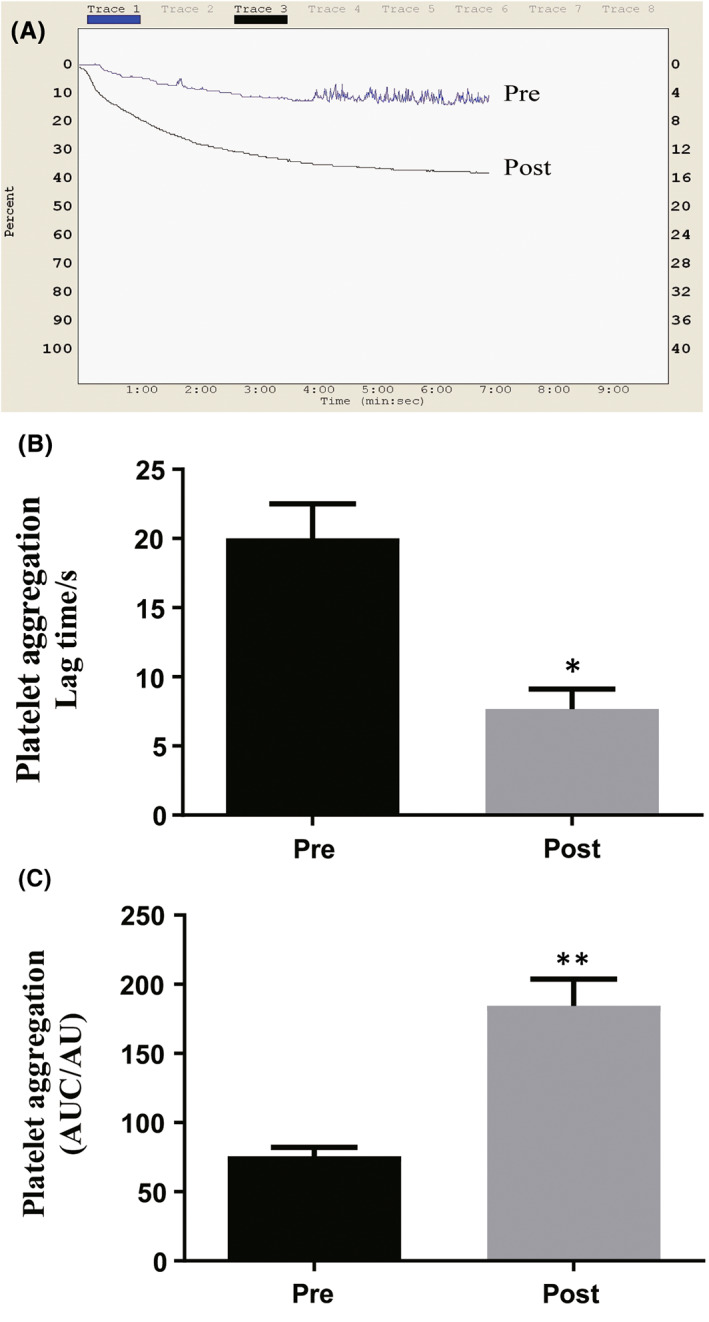
Sleep deprivation‐liberated small extracellular vesicles (EVs) promoted platelet aggregation in vitro. (A) Typical platelet aggregation tracing. Thrombin (10 μM) was used to induce platelet aggregation and the maximum aggregation rate of platelets was recorded within 7 min by turbidimetry using a LBY‐NJ4 aggregometer. (B) Platelet aggregation Lag time/s: Time of platelet aggregation after adding inducer. (C) Platelet aggregation (AUC/AU): Area of platelet aggregation in curve per unit time. Pre: pre‐sleep deprivation; Post: post‐sleep deprivation. Data are represented as means ± SEM, *p < 0.05, **p < 0.01.

To determine whether the prothrombotic effect of sleep deprivation‐liberated small EVs was related to alterations in platelet aggregation, we observed platelet aggregation induced by thrombin in vitro. We isolated plate‐rich plasma and incubated with small EV, followed by activating it by thrombin. As shown in Figure 4, sleep deprivation‐liberated small EVs clearly promoted thrombin‐induced rat platelet aggregation.

### 3.4 Sleep deprivation increased the levels of HMGB1 protein in small EVs

**FIGURE 5:**
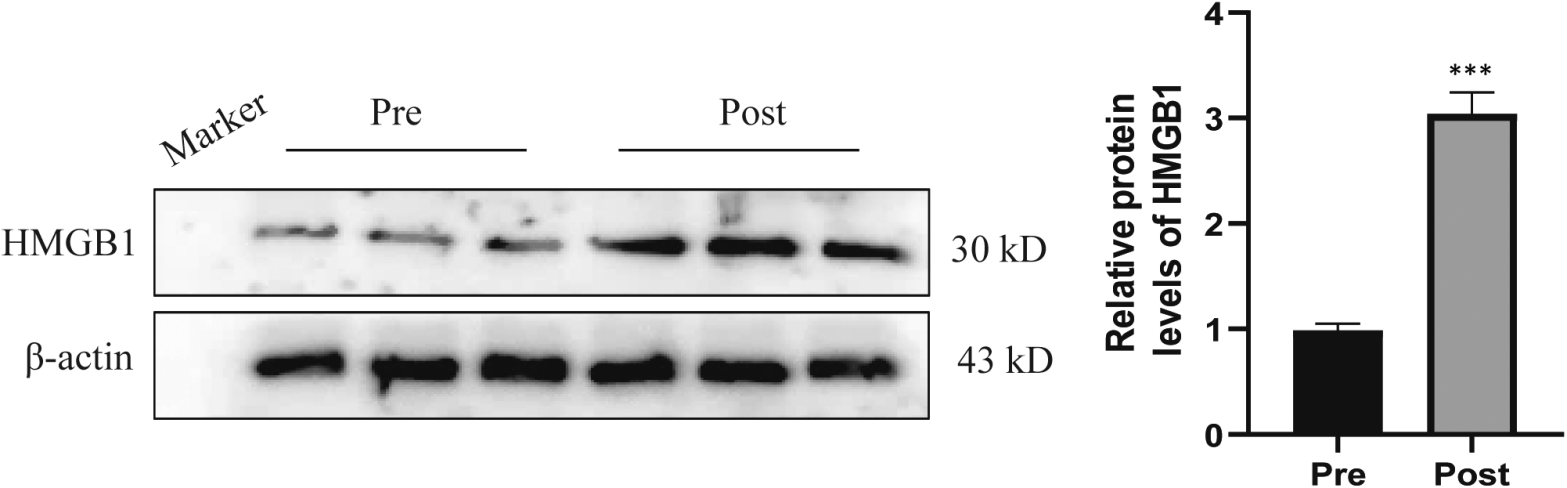
Sleep deprivation increased the levels of HMGB1 protein in small extracellular vesicles (EVs). Plasma samples were collected from 20 healthy participants before (pre) and after (post) sleep deprivation, and 100,000 g pellets were prepared. Protein content of the particles was analysed by Western blotting (*n* = 6) and the signals were quantified. Pre: pre‐sleep deprivation; Post: post‐sleep deprivation. Data are presented as mean ± SEM, ***p < 0.001.

Proteins from EVs are known to play important roles in various cellular functions. In this study, we evaluated the protein level of HMGB1, which is critical for regulating platelet activation, granule secretion, adhesion and spreading.^36^ As shown in Figure 5, the levels of HMGB1 protein increased on average 3 times in SD post‐liberated small EVs compared with pre‐liberated small EVs. The data suggested that the platelet aggregation and thrombus formation induced by SD‐liberated small EVs might be attributable to the elevated HMGB1 protein level in small EVs.

### 3.5 Sleep deprivation‐liberated small EVs demonstrate tropism to the liver

**FIGURE 6:**
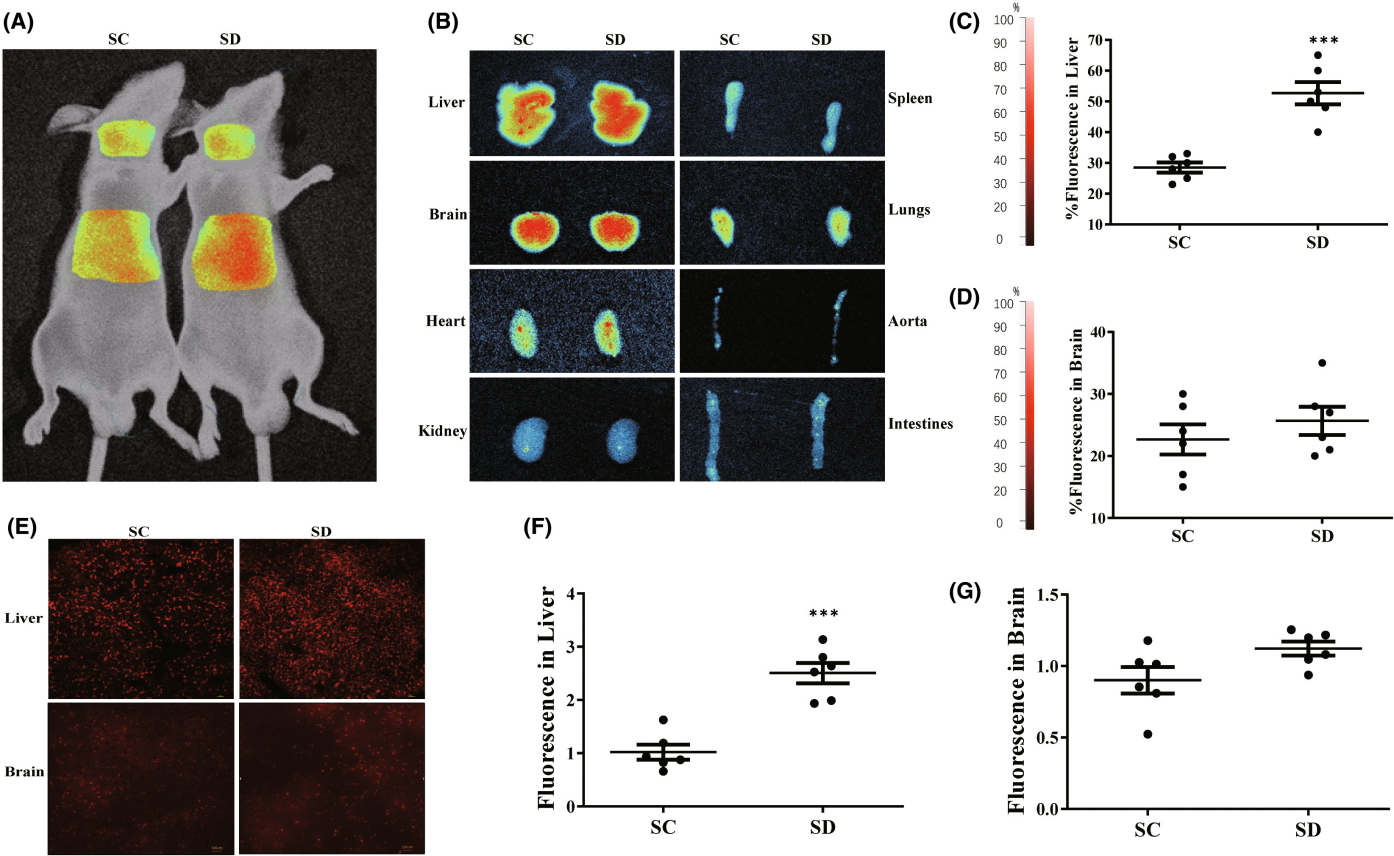
Sleep deprivation‐liberated small extracellular vesicles (EVs) demonstrate tropism to the liver. Intravital imaging of recipient mice treated intravenously with DiR‐labelled small EVs from rats undergoing one night of sleep deprivation and control mice. (A) Whole‐body fluorescence imaging of animals treated with a 200 μl dose of small EVs from 6 sleep deprivation or sleep control donor rats. Images were taken 12 h after tail vein injection. (B–D) Quantitative fluorescent imaging of liver, brain, heart and kidney taken from recipient mice receiving 200 μl dose of labelled small EVs from sleep deprived or sleep controlled donors. Images were taken at 30 min. Data are mean percentage fluorescence ± SD; ***p < 0.001; *n* = 6. (E–G) Rapid frozen section fluorescent imaging of liver and brain taken from recipient mice receiving 200 μl dose of labelled small EVs from sleep deprived or sleep controlled donors. ***p < 0.001; *n* = 6.

It is reported that small EVs communicate a wide range of information to target cells.^37^ Here, we hypothesized that small EVs participate in tissue crosstalk during sleep deprivation is the uptake of these vesicles in recipient tissues. To investigate the biodistribution of small EVs once they are liberated into circulation during sleep deprivation, we isolated small EVs from rats undergoing one night of sleep deprivation (SD group) and control rats (SC group) and labelled them with DiR. These labelled small EVs were then intravenously administered to recipient mice, which were then analysed by whole‐body intravital imaging. Eight hours after injection of a 150 μg dose of labelled small EVs, we detected fluorescence in mice receiving small EVs from SC and SD groups, respectively (Figure 6A), indicating that small EVs liberated into circulation by sleep deprivation more readily concentrate in tissues in the abdominal viscera, with a small amount in the brain. We then carried out repeat experiments in mice receiving labelled small EVs from a single donor at a one‐to‐one ratio and observed fluorescent signal in the liver and brain (Figure 6B). Importantly, we observed a significant increase in fluorescence in the livers of mice receiving small EVs from SD group versus control donors 8 h after injection (Figure 6B,C), indicating that small EVs liberated into circulation during sleep deprivation more readily localize in the liver.

## 4 DISCUSSION

Sleep is integral to life.^38^ Insufficient or disrupted sleep increases the risk of multiple pathological conditions, including acute myocardial infarction and coronary artery disease.^39^, ^40^ EVs emerge as comprehensive signalling entities mediating adaptive responses over large distances with widespread implications in physiology.^2^, ^41^ In this study, we investigated whether one night of sleep deprivation affects the level of EVs in the circulation with focus on small EVs. The results show that one night of sleep deprivation triggers release of small EVs into the circulation, which promote thrombosis formation and platelet activation, and more readily localize in the liver. These data suggested that small EVs may be implicated in long distance signalling during sleep deprivation‐mediated adaptation processes, and small EVs released here may increase the risk of thrombosis, probably by the increased HMGB1 protein levels.

Sleep deprivation is a condition that occurs if you do not get enough sleep. Sleep deficiency is a broader concept, which includes insomnia, acute sleep deprivation, sleep‐disordered breathing, restless legs syndrome, etc.^42^ Several reports have shown the alterations of EVs in sleep‐disordered condition. Abdelnaby et al. reported the plasma exosome might contribute to adipocyte metabolic dysfunction in patients with sleep‐disordered‐breathing (SDB), which is characterized by chronic intermittent hypoxia and sleep fragmentation.^43^ Plasma exosomes in obstructive sleep apnea syndrome (OSA) patients promote endothelial senescence.^44^ Also, increased microparticle levels in middle‐aged and elderly patients with insomnia may be involved in the pathogenesis of arteriosclerosis. But no studies have reported the effect of acute sleep deprivation on the alterations of circulating small EVs. Our findings provide preliminary evidence for the role of one night of SD on the levels of small EVs in the circulation. Our findings are consistent with previous reports of the significant rises of small EVs in healthy individuals after a single bout of physical exercise.^37^, ^45^ Given the fact that one night of SD and a single bout of exercise are both stressful conditions for the body, it is possible that small EV is an emerging factor in stress stimulation and probably play roles in intercellular communications subsequently.^46^ It has been reported that there is a trend for platelet‐derived microparticles to increase after cardiac stress,^47^ as well as a marked rise of microparticles in healthy individuals after a high‐fat meal,^48^ suggesting that larger EVs may have a physiological or pathophysiological function to promote or remove stress‐induced cellular by‐products.^49^ Looking at a total population of larger EVs by semi‐quantitative means, we did not find evidence for increased total levels of large EVs in the present study; however, this analysis is probably not powerful enough to resolve the dynamics of EV sub‐populations derived from distinct cells.

Examination of the αIIb integrin subunit indicated that platelet‐derived vesicles are among small EVs released in response to one night of SD. Platelets are known to produce pro‐coagulant EVs including exosomes as a result of different activation stimuli.^6^, ^50^ It should be noted that plasma processing affects platelet vesiculation and fractionation, which may contribute to the recovery of platelet EVs in MV and small EV fractions.^51^, ^52^ However, the pre‐analytical parameters were identical for all subjects in this study. Most likely, other cells such as endothelial cells, red blood cells, monocytes and neutrophils can release exosomes into the circulation. Further detailed studies are required to decipher the cellular source and the full complexity of small EVs released during sleep deprivation.

The interaction between small EVs and haematological system offered new insight into the coagulation and thrombosis. Disordered coagulation contributes to the formation of thrombosis in pathological conditions. Thrombosis is usually the final stage of atherosclerosis, and it can lead to severe results, including stroke, myocardial infarction and venous thromboembolism.^53^ Recently, proteomic study revealed that EVs in smoke people have a higher level of tissue factor, which may be correlated with thrombosis and atherosclerosis,^54^ providing evidences that high level of circulating EVs may trigger thrombosis.^55^ In the present study, we found that SD‐liberated small EVs increased thrombus formation and platelet aggregation in vivo and in vitro, further confirming that the amount of circulating EVs was increased by SD. In the following experiments, we found HMGB1 protein level in small EVs was elevated by sleep deprivation, suggesting one night of sleep deprivation promotes the release of small EVs along with the modification of small EV‐carried proteins. Platelet HMGB1 has a distinctive role in platelet thrombotic functions. After release from first‐responding platelets, HMGB1 attains concentrations that activate and aggregate other platelets and thereby, initiate a cascade of platelet thrombogenesis.^36^ The induction in EV level of HMGB1 by one night of SD suggests that this may be one mechanism by which such SD may promote platelet contributions to thrombosis and atherosclerosis.

Small EVs are heterogeneous nano‐sized vesicles with unique protein, lipid and nucleic acid compositions capable of eliciting different effects on recipient cells.^56^ This heterogeneity which might be coordinated during sleep disorder is a meaningful concept. Elemental to this is the directed transport of vesicles to specific tissues. In support of this we herein show, at least in transplanted rodents, that the biodistribution of vesicles following one night of SD differs from that seen in the absence of SD stimulus. Since blood flow in recipient animals would be equal, and the same amount of small EVs from SC and SD samples were injected, the observed difference in localization must be driven by factors intrinsic to the vesicles. The target specificity of small EVs is thought to be mediated by adhesion proteins such as integrins and tetraspanins on the surface of the vesicles.^57^ Further studies are needed to illustrate the components of small EVs. On the contrary, EV levels in the circulation depend on speed of the clearance, currently thought to involve direct receptor binding of liver or spleen phagocytes to phosphatidylserine or opsonization protein on the EVs.^58^ Another report shows that macrophages in the liver play a crucial role in the clearance of the exosomes from the circulation.^59^ In our study, we observed that small EVs liberated into circulation during sleep deprivation are more readily localize in the liver, suggesting the elevated small EV numbers induced by the sleep‐disordered stress might have a trend to be cleared in the liver to maintain the homeostasis of the body.

The present study has two limitations. First, the cellular source, comprehensive cargo alterations, targets and signalling components of SD‐induced small EVs were not validated to uncover the mechanism of their potential role as mediators of health‐injurious effects associated with sleep deprivation. Second, although we determined the subsequent distribution of small EVs, based on the fact that the release of small EVs in this study is an acute event, the dynamic changes of circulating small EVs remain to be elucidated.

## 5 CONCLUSIONS

In summary, we demonstrated one night of sleep deprivation triggers release of small EVs into the circulation, which promote thrombosis formation and platelet activation, and more readily localize in the liver. These data suggested that one night of sleep deprivation is a stress for small EV release, and EVs released here may increase the risk of thrombosis. Further, EVs may be implicated in long distance signalling during sleep deprivation‐mediated adaptation processes.

## AUTHOR CONTRIBUTIONS

**Chongyue Wang:** Conceptualization (equal); data curation (equal); formal analysis (equal); investigation (lead); methodology (lead); resources (lead); supervision (lead); validation (lead). **Lulu Li:** Data curation (equal); formal analysis (equal); investigation (lead); methodology (lead); resources (lead); validation (lead). **Chao Yang:** Project administration (equal); resources (equal). **Zhaoqiang Zhang:** Project administration (equal); resources (equal). **Xiao Li:** Project administration (equal); resources (equal). **Yun Wang:** Project administration (equal); resources (equal). **Xiang Lv:** Project administration (equal); resources (equal). **Xufeng Qi:** Project administration (equal); resources (equal). **Guohua Song:** Conceptualization (lead); data curation (lead); formal analysis (lead); funding acquisition (lead); investigation (equal); methodology (equal); project administration (equal); resources (equal); software (lead); supervision (lead); validation (equal); visualization (lead); writing – original draft (lead); writing – review and editing (lead).

## FUNDING INFORMATION

This study was supported by the National Natural Science Foundation of China (no. 81873517, 81670422, 81770240), Young Taishan Scholars Program of Shandong Province (tsqn20161045), and the academic promotion program of Shandong First Medical University (Shandong Academy of Medical Sciences) (no. 2019rc011), and National Innovation and Entrepreneurship Training Program for college students (no. 201910439009).

## CONFLICT OF INTEREST

We declare that we have no conflict of interest.

## Data Availability

The data that support the findings of this study are available from the corresponding author upon reasonable request.
